# Predictive factors for multidrug-resistant gram-negative bacteria among hospitalised patients with complicated urinary tract infections

**DOI:** 10.1186/s13756-018-0401-6

**Published:** 2018-09-14

**Authors:** Aina Gomila, Evelyn Shaw, Jordi Carratalà, Leonard Leibovici, Cristian Tebé, Irith Wiegand, Laura Vallejo-Torres, Joan M. Vigo, Stephen Morris, Margaret Stoddart, Sally Grier, Christiane Vank, Nienke Cuperus, Leonard Van den Heuvel, Noa Eliakim-Raz, Cuong Vuong, Alasdair MacGowan, Ibironke Addy, Miquel Pujol, on behalf of COMBACTE-MAGNET WP5- RESCUING Study

**Affiliations:** 1 Department of Infectious Diseases, Hospital Universitari de Bellvitge, Institut Català de la Salut (ICS-HUB), Feixa Llarga s/n, L’Hospitalet de Llobregat, 08907 Barcelona, Spain; 2 Spanish Network for Research in Infectious Diseases (REIPI RD12/0015), Instituto de Salud Carlos III, Madrid, Spain; 3 Institut d’Investigació Biomèdica de Bellvitge (IDIBELL), Feixa Llarga s/n, L’Hospitalet de Llobregat, 08907 Barcelona, Spain; 4 University of Barcelona, Barcelona, Spain; 5 Department of Medicine E, Beilinson Hospital, Rabin Medical Center, Petah Tikva; Sackler Faculty of Medicine, Tel Aviv University, Tel Aviv, Israel; 6 AiCuris Anti-infective Cures GmbH, Wuppertal, Germany; 7 UCL Department of Applied Health Research, University College London, London, UK; 8 Informatics Unit, Fundació Institut Català de Farmacologia, Barcelona, Spain; 9 Department of Medical Microbiology, Southmead Hospital, North Bristol NHS Trust, Bristol, UK; 10 Julius Center for Health Sciences and Primary Care, University Medical Center Utrecht, Utrecht, Netherlands

**Keywords:** Multidrug-resistance, Complicated urinary tract infection, Gram-negative bacteria, Predictive model of multidrug-resistance gram-negative bacteria

## Abstract

**Background:**

Patients with complicated urinary tract infections (cUTIs) frequently receive broad-spectrum antibiotics. We aimed to determine the prevalence and predictive factors of multidrug-resistant gram-negative bacteria in patients with cUTI.

**Methods:**

This is a multicenter, retrospective cohort study in south and eastern Europe, Turkey and Israel including consecutive patients with cUTIs hospitalised between January 2013 and December 2014. Multidrug-resistance was defined as non-susceptibility to at least one agent in three or more antimicrobial categories. A mixed-effects logistic regression model was used to determine predictive factors of multidrug-resistant gram-negative bacteria cUTI.

**Results:**

From 948 patients and 1074 microbiological isolates, *Escherichia coli* was the most frequent microorganism (559/1074), showing a 14.5% multidrug-resistance rate. *Klebsiella pneumoniae* was second (168/1074) and exhibited the highest multidrug-resistance rate (54.2%), followed by *Pseudomonas aeruginosa* (97/1074) with a 38.1% multidrug-resistance rate. Predictors of multidrug-resistant gram-negative bacteria were male gender (odds ratio [OR], 1.66; 95% confidence interval [CI], 1.20–2.29), acquisition of cUTI in a medical care facility (OR, 2.59; 95%CI, 1.80–3.71), presence of indwelling urinary catheter (OR, 1.44; 95%CI, 0.99–2.10), having had urinary tract infection within the previous year (OR, 1.89; 95%CI, 1.28–2.79) and antibiotic treatment within the previous 30 days (OR, 1.68; 95%CI, 1.13–2.50).

**Conclusions:**

The current high rate of multidrug-resistant gram-negative bacteria infections among hospitalised patients with cUTIs in the studied area is alarming. Our predictive model could be useful to avoid inappropriate antibiotic treatment and implement antibiotic stewardship policies that enhance the use of carbapenem-sparing regimens in patients at low risk of multidrug-resistance.

**Electronic supplementary material:**

The online version of this article (10.1186/s13756-018-0401-6) contains supplementary material, which is available to authorized users.

## Background

Urinary tract infections (UTIs) are one of the most common bacterial infections [1]. Complicated urinary tract infections (cUTIs), occurring in individuals with functional or structural urinary tract abnormalities, are a leading cause of hospital admissions, hospital-acquired infections, and antibiotic use [2].

The prevalence of cUTIs is difficult to assess accurately. Data from the most recent point prevalence survey of healthcare-associated infections (HAIs) in European acute care hospitals showed that UTI was the third most common cause, accounting for 19% of estimated 3.2 million overall cases of HAIs [3]. This figure, although huge, clearly underestimates the overall cUTI incidence in Europe because it did not include patients developing cUTIs in the community and in long-term care facilities (LTCFs). In LTCFs, cUTIs occur in more than one million patients annually [4]. Aging, comorbidities, and an increasing number of invasive urologic procedures for both diagnosis and treatment have been related to this high prevalence of cUTIs in the European population.

Antibiotic resistance has become a major healthcare problem in Europe and worldwide [5, 6]. Currently, multidrug-resistant (MDR) gram-negative bacteria (GNB) pose a threat in hospitals and nursing homes [7]. According to the recent Annual Report of the European Antimicrobial Resistance Surveillance Network (EARS-Net) [8], MDR rates showed large variations across Europe, being higher in southern and south-eastern Europe than in northern Europe. Patients with suspected cUTIs are frequently treated empirically with broad-spectrum antibiotics. Developing a model that helps select patients at high risk for MDR could be useful when choosing empirical antibiotic regimens and in antibiotic stewardship policies.

Considering the lack of contemporary data on hospitalised patients with cUTIs, we aimed to determine the prevalence of MDR among hospitalised patients with cUTIs in countries with high MDR-GNB prevalence and develop a predictive model to determine the risk of MDR-GNB infections, which would be useful to select more targeted antibiotic regimens avoiding the frequent treatment with broad-spectrum antibiotics.

## Methods

### Study design

The COMBACTE-MAGNET, WP5 RESCUING Study was a multicenter, retrospective, observational cohort study including hospitalised patients with cUTI from January 2013 to December 2014. Data was collected from patients who were diagnosed with cUTI as the primary cause of hospitalisation and from patients who were hospitalised for other reasons but who developed cUTIs during their hospitalization [9]. This study conformed to the STROBE guidelines for reporting observational studies [10].

### Setting and patients

The study was conducted in Bulgaria (2 hospitals), Greece (2 hospitals), Hungary (3 hospitals), Israel (3 hospitals), Italy (3 hospitals), Romania (2 hospitals), Spain (3 hospitals) and Turkey (2 hospitals). Patients were identified by searching for the appropriate International Classification of Diseases (ICD)-9 Clinical Modification (CM) or ICD-10 CM Codes [11, 12] at discharge from hospital (diagnoses are detailed in Additional file 1). All patients who met the criteria for cUTI were selected for data collection. In order to avoid selection bias, each hospital included 50 to 60 consecutive patients with cUTI until achieving the total estimated sample size of 1000 patients.

Complicated urinary tract infection inclusion criteria followed the Food and Drug Administration (FDA) guidance on cUTI [13], and consisted on:

- Patients with UTI and at least one of the following underlying conditions: a) indwelling urinary catheter; b) urinary retention (at least 100 mL of residual urine after voiding); c) neurogenic bladder; d) obstructive uropathy (e.g., nephrolithiasis, fibrosis); e) renal impairment caused by intrinsic renal disease (estimated glomerular filtration rate < 60 mL/min); f) renal transplantation; g) urinary tract modifications, such as an ileal loop or pouch; or h) pyelonephritis.
- And at least one of the following signs or symptoms: a) chills or rigors associated with fever or hypothermia (temperature > 38 °C or < 36 °C); b) flank pain (pyelonephritis) or pelvic pain (cUTI); c) dysuria, urinary frequency, or urinary urgency; or d) costovertebral angle tenderness on physical examination.
- And urine culture with ≥10^5^ colony-forming units/mL of uropathogen (no more than two species) or;
- At least one blood culture growing possible uropathogens (no more than two species) with no other evident site of infection.

The exclusion criteria were as follows: a) patients aged < 18 years, b) diagnosis of prostatitis according to FDA guidance, c) polymicrobial infections including *Candida* spp., d) polymicrobial infections including more than two bacterial species, or e) cUTI with *Candida* spp. as sole uropathogen, d) patients with uncomplicated cystitis.

If a patient had more than one episode of cUTI during the same hospitalisation, only the first episode was included.

### Data collection and validation

Data on demographic characteristics, comorbidities, place of acquisition of infection, signs and symptoms of infection, laboratory and microbiology, imaging tests, management of infection including antibiotic therapy and interventional procedures, details of discharge and outcome of infection, including death if applicable, were reviewed by professionals who received web-database training sessions. For data collection, an access-controlled web-based electronic case report form was used. At each site, a screening log was kept of the patients with infections detected according to the ICD codes, detailing the excluded patients and the reasons for exclusion. To confirm data quality, study sites were monitored and audited by a contract research organization (CRO) from Utrecht, Netherlands.

### Definitions

Acquisition of cUTI in a medical care facility was considered if it was:

- Hospital-acquired: if it started ≥48 h after hospital admission.
- Healthcare-associated: if it was detected at hospital admission or within the first 48 h of hospitalization, with the patient fulfilling any of the following criteria: 1) receiving intravenous therapy, wound care, or specialized nursing care at home in the previous 30 days; 2) admission in the hospital or haemodialysis ward or receiving intravenous chemotherapy in the previous 30 days; 3) hospitalization for ≥2 days in the previous 90 days; 4) residence in a long-term care facility; 5) underwent invasive urinary procedure within the previous 30 days; or 6) having a long-term indwelling urinary catheter.

We used the following categories for cUTIs:

- UTI related to indwelling urinary catheterization, including long-term, short-term, or intermittent catheterization
- Pyelonephritis with no other urinary tract modification, defined as sepsis, flank pain or costovertebral angle tenderness
- UTI related to anatomical urinary tract modification, including any urinary diversion procedure, nephrostomy or stents, or renal transplants
- UTI related to obstructive uropathy, including any obstruction intrinsic or extrinsic to the urinary tract, such as lithiasis, tumor, ureteral herniation, or prostate hyperplasia
- UTI related to other events that do not fall under any other category

Multidrug resistance was defined according to an international expert proposal by Magiorakos et al. [14], as non-susceptibility to at least one agent in three or more antimicrobial categories (extended-spectrum penicillins, carbapenems, cephalosporins, aminoglycosides, and fluoroquinolones). Extensively drug-resistance (XDR) was defined as non-susceptibility to at least one agent in all but two or fewer antimicrobial categories (i.e., bacterial isolates remaining susceptible to only one or two categories) tested for a determined microorganism.

### Outcomes

The primary outcome was the presence of MDR, as previously defined.

Secondary outcomes included the following:

- Estimation of the MDR prevalence in each country and participating hospital
- Definition of the most prevalent microbiology according to source of infection
- Assessment of the resistance rate of the main GNB to the different antimicrobial classes

### Statistical methods

The chi-square or Fisher’s exact test was used to compare categorical data, and Student’s t-test or the Mann-Whitney U test to compare continuous data, as appropriate. The quantile-quantile normality plot and Kolmogorov-Smirnov test were used to assess whether a continuous variable was normally distributed.

#### Predictive model of MDR in patients with cUTI

Countries and hospitals presented a non-homogeneous MDR baseline risk. To account for such variations, a mixed-effects logistic regression model to predict the risk of MDR in patients with cUTIs, including all different epidemiological and clinical variables, was built using hospitals as clusters. First, a stepwise selection method based on the Akaike Information Criterion was performed to identify variables that explained the bulk of MDR infections. Adequacy of the final model was assessed by collinearity, influential observations, and residuals. To evaluate discrimination properties, the Hosmer-Lemeshow goodness-of-fit test was used. Moreover, the bootstrapping resampling method was used to improve the robustness of estimated standard errors. Results were given as odds ratios (OR) and 95% confidence intervals (95% CI). All tests were two-tailed, and a *p*-value of < 0.05 was considered statistically significant.

All data were analyzed using R software (2017). R Foundation for Statistical Computing, Vienna, Austria.

## Results

### Patients’ epidemiological characteristics and univariate analysis of MDR-GNB

Fifty-two cases were excluded due to lack of information on the presence of MDR, leaving a final sample of 948 patients. Among them, 1074 bacterial isolates were obtained.

The patients’ clinical characteristics are shown in Table 1. Females comprised 56%, the mean age was 65.8 ± 18.2 years, 34.4% were admitted due to conditions other than cUTIs, 17.4% came from LTCFs, and 46% were functionally dependent. Factors associated with MDR by univariate analysis were male gender, admission due to reasons other than cUTIs, residing in LTCF, dependent functional capacity, UTI within the previous year, antibiotic treatment within the previous 30 days, acquisition of cUTI in a medical care facility, and presence of an indwelling urinary catheter.

**Table 1:** Patients’ epidemiological characteristics and univariate analysis of multidrug-resistance in gram-negative bacteria

	Entire Cohort (*n* = 948)	Susceptible (*n* = 691)	MDR (*n* = 257)	*p*-Value
Male gender, n (%)	420 (44.3)	270 (39.1)	150 (58.4)	< 0.001
Mean age (SD), years	65.8 (18.2)	65.6 (18.6)	66.5 (16.8)	0.526
Elective admission, n (%)	141 (14.9)	97 (14)	44 (17.1)	0.236
Admission reason: conditions other than cUTI, n (%)	326 (34.4)	214 (31)	112 (43.6)	< 0.001
Place of residency: long-term care facility, n (%)	165 (17.4)	98 (14.2)	67 (26.1)	< 0.001
Underlying disease, n (%)				
Acute myocardial infarction	79 (8.3)	56 (8.1)	23 (8.9)	0.676
Congestive heart failure	182 (19.2)	134 (19.4)	48 (18.7%)	0.804
Peripheral vascular disease	70 (7.4)	55 (8)	15 (5.8)	0.267
Cerebrovascular disease	182 (19.2)	122 (17.7)	60 (23.3)	0.048
Dementia	130 (13.7)	93 (13.5)	37 (14.4)	0.709
Chronic pulmonary disease	135 (14.2)	91 (13.2)	44 (17.1)	0.122
Connective tissue disease	21 (2.2)	15 (2.2)	6 (2.3)	0.879
Peptic ulcer	46 (4.9)	34 (4.9)	12 (4.7)	0.873
Diabetes mellitus	250 (26.4)	186 (26.9)	64 (24.9)	0.531
Chronic kidney disease	263 (27.7)	191 (27.6)	72 (28)	0.909
Hemiplegia	86 (9.1)	58 (8.4)	28 (10.9)	0.233
Leukaemia	9 (0.9)	6 (0.9)	3 (1.2)	0.673
Lymphoma	13 (1.4)	11 (1.6)	2 (0.8)	0.338
Chronic liver disease	50 (5.3)	35 (5.1)	15 (5.8)	0.637
Solid tumour	114 (12.3)	75 (11.1)	39 (15.4)	0.075
Metastatic tumour	47 (5)	35 (5.1)	12 (4.7)	0.803
Valvulopathy	88 (9.3)	69 (10)	19 (7.4)	0.221
HIV infection	10 (1.1)	8 (1.2)	2 (0.8)	0.611
Charlson index ≥ 3, n (%)	418 (44.1)	299 (43.3)	119 (46.3)	0.403
Organ transplant, n (%)	65 (6.9)	45 (6.5)	20 (7.8)	0.492
Immunosuppression, n (%)	94 (9.9)	64 (9.3)	30 (11.7)	0.270
Steroids, n (%)	68 (7.2)	46 (6.7)	22 (8.6)	0.313
Functional capacity: dependent, n (%)	436 (46.1)	298 (43.3%)	138 (53.9)	0.003
Prior UTI (within the previous year), n (%)	247 (26.1)	167 (24.2)	80 (31.2)	0.027
Prior antibiotics (within the previous 30 days), n (%)	190 (20.1)	120 (17.4)	70 (27.6)	0.001
Prior quinolone	64 (6.8)	38 (5.5)	26 (10.2)	0.010
Prior Penicillin	55 (5.8)	35 (5.1)	20 (7.9)	0.103
Prior cephalosporin	42 (4.4)	27 (3.9)	15 (5.9)	0.188
Prior Carbapenem	22 (2.3)	10 (1.4)	12 (4.7)	0.003
Prior other antibiotics	51 (5.4)	31 (4.5)	20 (7.9)	0.042
Acquisition in a medical care facility, n (%)	410 (43.2)	244 (35.3)	166 (64.6)	< 0.001
Source of cUTI, n (%)				
Indwelling urinary catheterisation	308 (32.5)	189 (27.4)	119 (46.3)	< 0.001
Pyelonephritis with normal tract anatomy	196 (20.7)	164 (23.7)	32 (12.5)	< 0.001
Obstructive uropathy	152 (16)	114 (16.5)	38 (14.8)	0.523
Urinary tract diversion	84 (8.9)	64 (9.3)	20 (7.8)	0.476
Other	208 (21.9)	160 (23.2)	48 (18.7)	0.139
Shock/severe sepsis, n (%)	140 (15.9)	104 (16.2)	36 (14.9)	0.635

*MDR* multidrug resistance, *SD* standard deviation, *cUTI* complicated urinary tract infection, *HIV* human immunodeficiency virus, *UTI* urinary tract infection

### Most frequent bacterial aetiology and patterns of antimicrobial resistance

Of all bacterial isolates (*n* = 1074), the most frequent was *Escherichia coli*, isolated in 52% of samples, followed by *Klebsiella pneumoniae* in 15.6%, *Pseudomonas aeruginosa* in 9%, *Proteus mirabilis* in 7.3%, and *Enterococcus* spp. in 3.2%. Only these 5 bacteria were evaluated due to their clinical significance. *Escherichia coli* was mainly related to pyelonephritis with normal urinary tract (76.5%), while *K. pneumoniae* was more frequently associated with urinary tract diversion (22.6%). *Pseudomonas aeruginosa, P. mirabilis* and *Enterococcus* spp. were significantly related to the presence of an indwelling urinary catheter (18.8%, 25.6% and 5.8% respectively) (Table 2).

**Table 2:** Most frequent bacterial aetiology of complicated urinary tract infections according to source of infection (sources = 948, isolations = 1074)

Source (*n* = 948)	*E. coli* *n* = 559 (52%)	*K. pneumoniae* *n* = 168 (15.6%)	*P. aeruginosa* *n* = 97 (9%)	*P. mirabilis* *n* = 79 (7.3%)	*Enterococcus* spp. *n* = 34 (3.2%)
Indwelling urinary catheterisation (*n* = 308), n (%)	124 (40.3%)	63 (20.4%)	58 (18.8%)	40 (25.6%)	18 (5.8%)
Pyelonephritis with normal tract anatomy (*n* = 196), n (%)	150 (76.5%)	25 (12.7%)	4 (2.0%)	13 (6.6%)	0 (0.0)
Obstructive uropathy (*n* = 152), n (%)	98 (64.4%)	26 (17.1%)	12 (7.9%)	11 (7.2%)	5 (3.3%)
Urinary tract diversion (*n* = 84), n (%)	48 (57.1%)	19 (22.6%)	10 (11.9%)	2 (2.4%)	4 (4.8%)
Others (*n* = 208), n (%)	139 (66.8%)	35 (16.8%)	13 (6.2%)	13 (6.2%)	7 (3.4%)

*E. coli*, *Escherichia coli*; *K. pneumoniae*, *Klebsiella pneumoniae*; *P. aeruginosa*, *Pseudomonas aeruginosa*; *P. mirabilis*, *Proteus mirabilis*; *Enterococcus* spp., Enterococcus species. First column include all sources of infection (*n* = 948), and first raw include the five more frequent bacteria taking as denominator the total number of isolations (*n* = 1074). All other isolates up to the total number are not included in the table. Denominators in central boxes are the total number of each row (sources)

**Fig. 1:**
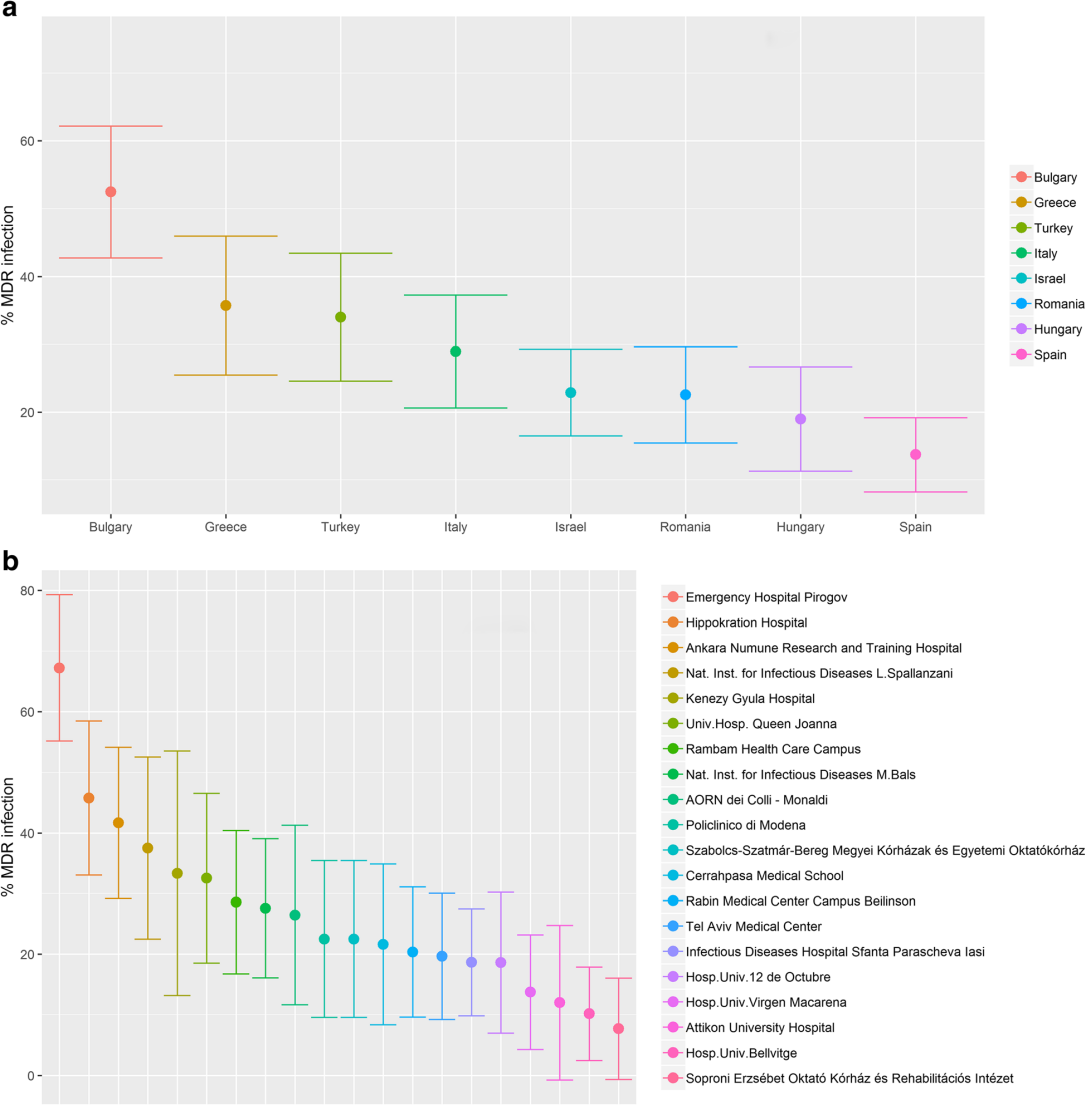
Cumulative multidrug-resistant gram-negative bacteria incidence with 95% confidence interval by country (**a**) and by hospital (**b**). Figure 1 shows the cumulative incidence with 95% confidence interval of multidrug-resistant (MDR) gram-negative bacteria in complicated urinary tract infection observed in each participating country (**a**) and in each participating hospital (**b**). Hosp. Univ. 12 de Octubre: Hospital Universitario 12 de Octubre, Hosp. Univ. Virgen Macarena: Hospital Universitario Virgen de la Macarena, Hosp. Univ. Bellvitge: Hospital Universitari de Bellvitge

Significant differences in MDR rate occurred between the different participating hospitals, ranging from < 20% in some countries such as Hungary and Spain to almost 60% in other countries such as Bulgaria and Greece (Fig. 1a). The MDR rates by hospital varied in accordance with the country’s trend (Fig. 1b).

The antimicrobial resistance patterns according to the most frequent GNB are shown in Table 3. *Escherichia coli* had a fluoroquinolone resistance rate of 39.5%, a third-generation cephalosporin (3GC) resistance rate of 24.2% and a MDR rate of 14.5%. *Klebsiella pneumoniae* exhibited the highest MDR rate (54.2%), followed by *P. aeruginosa* (38.5%) and *P. mirabilis* (24.1%). By antibiotic class, fluoroquinolones had the highest resistance rates (39.5% in *E. coli*, 56.5% in *K. pneumoniae*, 42.1% in *P. aeruginosa*, and 55.7% in *P. mirabilis*), followed by 3GC and aminoglycosides (Table 3). Resistance to carbapenems was 32.6% in *P. aeruginosa*, 19.6% in *K. pneumoniae* and 2.3% in *E. coli*.

**Table 3:** Patterns of antimicrobial resistance to main antibiotic groups by the four most frequent gram-negative bacteria

	AMG-R n (%)	FQ-R n (%)	3GC-R n (%)	P/T-R n (%)	CARB-R n (%)	MDR n (%)	XDR n (%)
*E. coli* (*n* = 559)	108 (19.3)	221 (39.5)	135 (24.2)	57 (10.2)	13 (2.3)	81 (14.5)	2 (0.4)
*K. pneumoniae* (*n* = 168)	77 (45.8)	95 (56.5)	98 (58.3)	64 (38.1)	33 (19.6)	91 (54.2)	23 (13.7)
*P. aeruginosa* (*n* = 97)	36 (37.9)	40 (42.1)	47 (49.5)	30 (31.6)	31 (32.6)	36 (38.5)	16 (16.8)
*P. mirabilis* (*n* = 79)	29 (36.7)	44 (55.7)	20 (25.4)	9 (11.4)	4 (5.0)	19 (24.1)	1 (1.3)

*AMG-R* aminoglycoside-resistant, *FQ-R* fluoroquinolone-resistant, *3GC-R* third-generation cephalosporin-resistant, *P/T-R* piperacillin/tazobactam-resistant, *CARB-R* carbapenem-resistant, *MDR* multidrug-resistant, *XDR* extensively drug-resistant, *E. coli*, *Escherichia coli*; *K. pneumoniae*, *Klebsiella pneumoniae*; *P. aeruginosa*, *Pseudomonas aeruginosa*; *P. mirabilis*, *Proteus mirabilis*

### Predictive model of MDR-GNB in patients with cUTIs

**Fig. 2:**
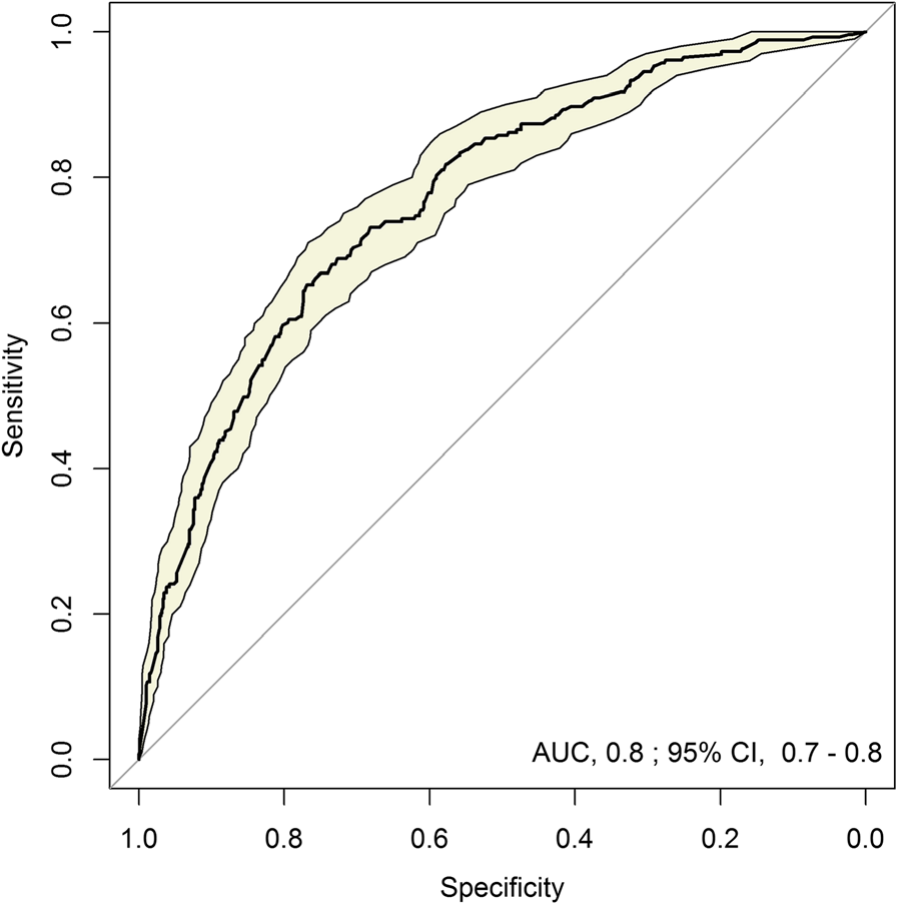
Receiver operating characteristic curve of the predictive model of multidrug-resistance in gram-negative bacteria. Figure 2 shows the evaluation of the discriminative power of the mixed-effects logistic regression predictive model for multidrug-resistant gram-negative bacteria among patients with complicated urinary tract infection by the receiver operating characteristic curve using observed multidrug-resistance incidence as the gold standard. AUC, area under the curve; CI, confidence interval

**Fig. 3:**
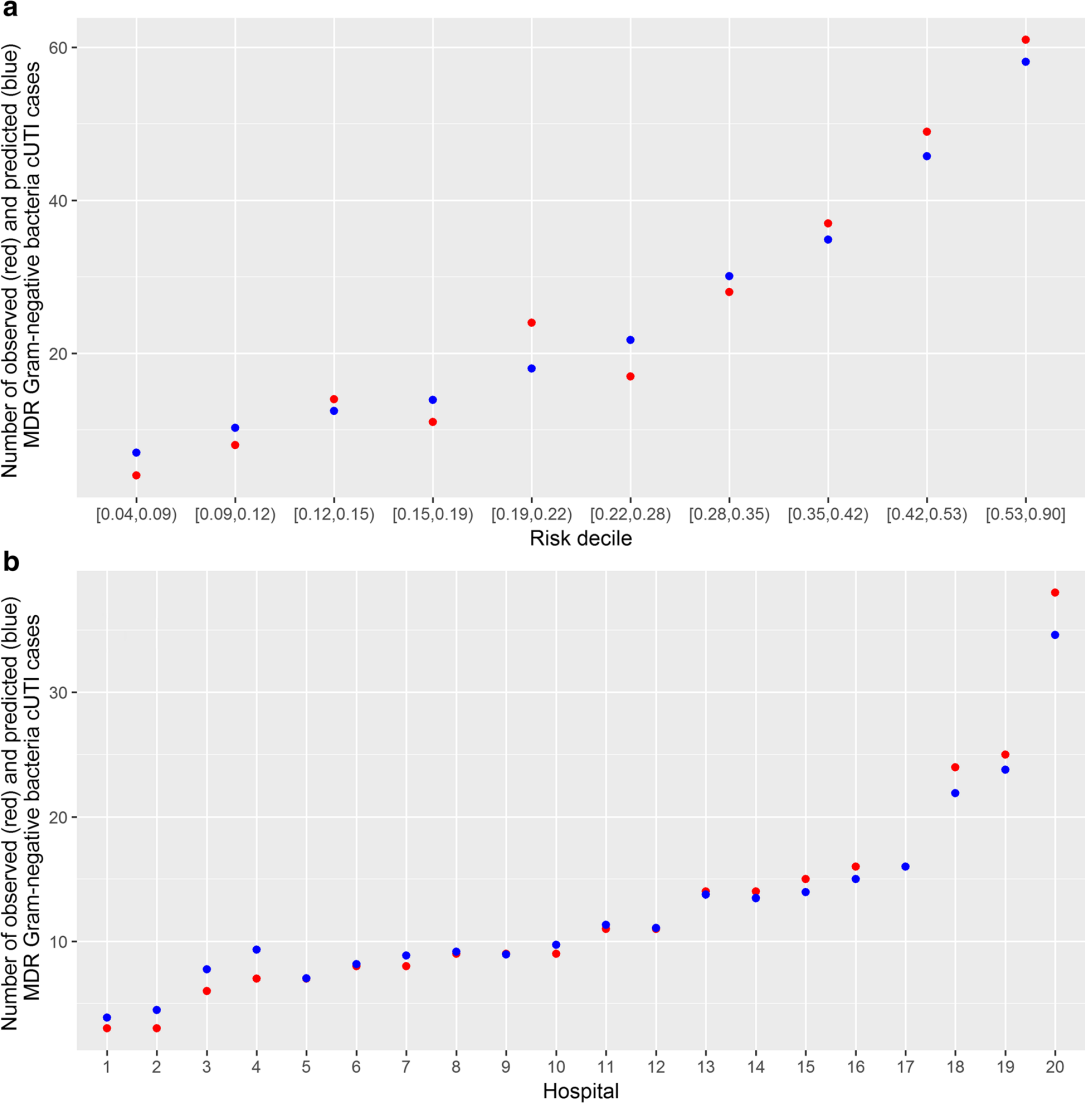
Observed versus predicted multidrug-resistant gram-negative bacteria risk, stratified by deciles of predicted risk (**a**) and by hospital (**b**). **a** shows the observed to expected events by probability deciles and (**b**) shows the observed to expected events by hospital. Events are defined as multidrug-resistant (MDR) gram-negative bacteria complicated urinary tract infections. Hospitals included in (**b**) are: 1. Soproni Erzsébet Oktató Kórház és Rehabilitációs Intézet, 2. Hospital Universitari de Bellvitge, 3. Attikon University Hospital, 4. Hospital Universitario Virgen de la Macarena, 5. Hospital Universitario 12 de Octubre, 6. Infectious Diseases Hospital Sfanta Parascheva Iasi, 7. Tel Aviv Medical Center, 8. Rabin Medical Center Campus Beilinson, 9. Cerrahpasa Medical School, 10. Szabolcs-Szatmár-Bereg Megyei Kórházak és Egyetemi Oktatókórház, 11. Policlinico di Modena, 12. AORN dei Colli – Monaldi, 13. National Institute for Infectious Diseases Matei Bals, 14. Rambam Health Care Campus, 15. University Hospital Queen Joanna, 16. Kenezy Gyula Hospital, 17. National Institute for Infectious Diseases Lazzaro Spallanzani, 18. Ankara Numune Research and Training Hospital, 19. Hippokration Hospital, 20. Emergency Hospital Pirogov

Identified predictive factors for MDR risk are reported in Table 4. The resulting equation and an illustrative example for calculating MDR-GNB risk are described in Additional file 1. The proposed model had good discrimination for MDR prediction, with a 0.80 statistic (area under the receiver operating characteristic curve) (Fig. 2). Calibration was also excellent, with a good observed/expected ratio of MDR risk by deciles of predicted risk (Fig. 3a) and by hospital **(**Fig. 3b**).**

**Table 4:** Predictive model of multidrug-resistant gram-negative bacteria in patients with complicated urinary tract infection: a mixed-effects logistic regression model

Factors	OR	95% CI	*p*-Value
(Intercept)	0.1	0.06–0.16	< 0.001
Gender (male)	1.66	1.20–2.29	0.002
Acquisition in a medical facility	2.59	1.80–3.71	< 0.001
Indwelling urinary catheter	1.44	0.99–2.10	0.06
UTI within the previous year	1.89	1.28–2.79	0.001
Antibiotics within the previous 30 days	1.68	1.13–2.50	0.011

*OR* odds ratio, *CI* confidence interval, *UTI* urinary tract infection

Potential predictors included in the predictive model were age, sex, source of infection, place of residency, functional capacity score, personal history of myocardial infarction, congestive heart failure, peripheral vascular disease, cerebrovascular disease, dementia, chronic pulmonary disease, ulcer disease, diabetes mellitus, chronic kidney disease, hemiplaegia, solid tumor, liver disease, metastatic tumor, Charlson score, infection acquisition site, presence of indwelling urinary catheter, urinary retention, organ transplant, kidney organ transplant, immunosuppressive therapy, active chemotherapy, corticosteroid therapy, UTI within the previous year, previous 30-day antibiotic treatment (including previous treatment with quinolones, penicillins, cephalosporins, carbapenems, and other antibiotics), infection severity, neurogenic bladder, obstructive uropathy, other urinary tract modification, and chronic renal impairment

The factors that best predicted the bulk of MDR presence were male gender (odds ratio [OR], 1.66; 95% confidence interval [CI], 1.20–2.29), acquisition of cUTI in a medical care facility (OR, 2.59; 95% CI, 1.80–3.71), presence of an indwelling urinary catheter (OR, 1.44; 95% CI, 0.99–2.10), having a UTI within the previous year (OR, 1.89; 95% CI, 1.28–2.79), and antibiotic treatment within the previous 30 days (OR, 1.68; 95% CI, 1.13–2.50) (Table 4).

## Discussion

This large, multicenter, retrospective cohort study of hospitalised patients with cUTIs provides a comprehensive update about antibiotic resistance in countries with high MDR incidence. In this cohort, *K. pneumoniae* had the highest MDR rate among all the GNB analysed, and fluoroquinolones had the highest resistance rates. We developed a model to predict the risk of cUTIs caused by MDR organisms, in order to avoid inappropriate treatment and help establish antibiotic stewardship policies.

In our cohort, *E. coli* continues to be the most frequent cause of cUTI, as previously observed [15, 16]. Although it was associated with low MDR levels, it showed a fluoroquinolone resistance rate of almost 40% and a 3GC resistance rate of 24%. Previous studies already described an increased resistance rate of *E. coli* to fluoroquinolones and trimethoprim-sulfamethoxazole, precluding their use as empiric treatment in mild and severe infections [16, 17]. Similar to our results, the Study for Monitoring Antimicrobial Resistance Trends (SMART) in the United States showed a 35% resistance rate of *E. coli* to ciprofloxacin [18]. This high fluoroquinolone resistance rate contrasts with the 20% reported by the EARS-Net in 2016 [8]. However, the rate was obtained by including northern European countries that had low antimicrobial resistance rates. On the contrary, the south-eastern countries showed rates similar to those observed in our study. Besides, the EARS-Net included only invasive isolates, a sample profile quite different to ours. MDR rates similar to our results were also observed in the Asia-Pacific region [19].

In our cohort, *K. pneumoniae* was the second most frequent microorganism, showing a remarkably carbapenem-resistance rate of almost 20% and having ileal loop or urinary diversion as the most frequent source of infection. The countries with the highest rate of carbapenem-resistant *K. pneumoniae* were Greece and Turkey, while those with the lowest were Spain and Hungary. This study did not analyse the type of resistance mechanisms present in Enterobacteriaceae; nevertheless, phenotypic resistance to carbapenems commonly results from acquiring carbapenemases that affect even the latest generations of penicillins and cephalosporins, in addition to other antibiotic families such as aminoglycosides and fluoroquinolones. The European survey of carbapenemase-producing Enterobacteriaceae (EuSCAPE), performed in 2013–2014 in Europe, Turkey, and Israel, showed that *K. pneumoniae* and *E. coli* produced carbapenemases, mainly KPC-type and OXA-48-like, in the countries represented in our study [20]. However, 29% of *K. pneumoniae* isolates had unidentified mechanisms of carbapenem resistance, and almost 10% of *K. pneumoniae* isolates were resistant to all antibiotics tested, consistent with our findings.

*P. aeruginosa* isolates showed a carbapenem-resistance rate that reached 32%. In this case, the presence of a urinary catheter was the most frequently associated factor. Countries with the greatest rates of carbapenem-resistant *P. aeruginosa* included Italy and Turkey, while those with the lowest rates were Israel and Hungary. The mechanisms of MDR in *P. aeruginosa* have been related to the production of cephalosporinases, combined with mutations that decrease carbapenem permeability of the bacterial cell wall [21–23]. The selective antibiotic pressure caused by broad-spectrum antibiotics favours the emergence of MDR strains, and once it is produced, its reversion is very slow [24].

We have developed and internally validated a clinical predictive model for hospitalised patients with suspected cUTIs that helps determine the risk of MDR-GNB infections, considering the country’s baseline risk. This model may be useful in reducing inappropriate empirical antibiotic treatment that leads to poor clinical outcomes in these patients [25]. It may also help implement antibiotic stewardship programs that enhance the use of carbapenem-sparing antibiotic regimens in patients at low risk for MDR [24, 26]. The severity of infection based on physician’s clinical judgement and severity scores needs to be assessed, since non-severe cUTI will probably benefit more from receiving treatment based on susceptibility testing [27]. Importantly, however, more severe cases with potentially serious consequences of treatment failure could benefit from applying our model.

The most reliable factor that predicted MDR was the acquisition of cUTIs in medical care facilities, mostly LTCFs. Most patients admitted to LTCFs are old, have comorbidities, and are functionally dependent. These patients frequently receive repeated courses of antibiotics for various reasons, including cUTIs. Thus, LTCFs have been identified as important reservoirs of MDR-GNB [28]. Besides patients having had a UTI within the previous year and having received antibiotics within the previous month, other predictive factors for MDR identified by our model have been also described by other authors [29, 30]. All of them reflect high cumulative exposure to antibiotics and, consequently, selection of MDR endogenous flora.

Male UTI is usually considered complicated due to the more complex urinary tract anatomy. This implies longer antibiotic treatments and frequent relapse of infection, resulting in repeated antibiotic exposure and higher risk of MDR [31].

The presence of a urinary catheter has been associated with a higher risk of UTI [32, 33] and infections caused by microorganisms with higher intrinsic resistance, such as *P. aeruginosa* and *Enterococcus* spp. [1]. Our study reaffirmed this observation since *P. aeruginosa* was significantly associated with urinary catheter use. The catheter inhibits the defence mechanisms of the urinary tract epithelium against bacteria and facilitates the rapid invasion of the bladder by microorganisms colonizing the device. The urinary catheter also promotes the development of bacterial biofilm, where antibiotics do not achieve significant concentrations [34].

The main limitation of this study is that the model has been validated in a group of hospitals from south and eastern Europe, Turkey and Israel and the results may not be generalizable to other populations. Therefore, further external validation is necessary to confirm our results. Also, the retrospective design and approach for identifying cases could have led to underestimate non-severe cases occurring in patients admitted due to other reason than cUTI and who developed cUTI during the hospitalisation. On the other hand, difficult to treat MDR-GNB cUTIs could have been more easily identified. The main strength of the study is its large-scale, multicenter, and multinational design including 948 patients and the case-validation system. Furthermore, the effect of possible differences in MDR baseline risk by each hospital on the main outcome was considered to create the predictive model.

## Conclusions

The current high rate of MDR-GNB infections among hospitalised patients with cUTIs is alarming in south and eastern Europe, Turkey and Israel. A high MDR rate has been observed among *K. pneumoniae* and *P. aeruginosa* isolates. Our study developed a predictive model that could be useful in determining the risk for MDR-GNB cUTI, with the purpose of targeting patients at high risk with broad-spectrum antibiotics and guiding the implementation of antibiotic stewardship policies that enhance the use of carbapenem-sparing antibiotic regimens in patients at low risk for MDR-GNB.

## Additional file

Additional file 1: Supplementary material. (DOCX 24 kb)

## Abbreviations

95%CI: 95% confidence interval
CRO: Contract research organization
cUTIs: Complicated urinary tract infections
EARS-Net: European Antimicrobial Resistance Surveillance Network
EuSCAPE: European survey of carbapenemase-producing Enterobacteriaceae
FDA: Food and Drug Administration
GNB: Gram-negative bacteria
HAIs: Healthcare-associated infections
ICD-9 (− 10) CM: International Classification of Diseases-9 (− 10) Clinical Modification
KPC: *Klebsiella pneumoniae* carbapenemase
LTCFs: Long-term care facilities
MDR: Multidrug-resistance
OR: Odds ratio
OXA: Oxacillinase
SMART: Study for Monitoring Antimicrobial Resistance Trends
STROBE: Strengthening the Reporting of Observational studies in Epidemiology
UTIs: Urinary tract infections
XDR: Extensively drug-resistance
