# Supplementary material for: Predictive factors for multidrug-resistant gram-negative bacteria among hospitalised patients with complicated urinary tract infections
Source: Antimicrob Resist Infect Control. 2018 Sep 14;7:111. doi: 10.1186/s13756-018-0401-6 (PMC6137881; doi:10.1186/s13756-018-0401-6)
Supplement: Supplementary file 1 — Supplementary material. (DOCX 24 kb) [file 13756_2018_401_MOESM1_ESM.docx]

**Supplementary material**

**Description of ICD-9 CM Codes and ICD-10 CM Codes**

**International Classification of Diseases ICD-9 Data**
http://icd9data.com/2015/volume1/580-629/default.htm

| **ICD-9 CM Codes** | **Disease** |
| --- | --- |
| 590.1 | Acute pyelonephritis |
| 590.10 | Acute pyelonephritis without lesion of renal medullary necrosis |
| 590.11 | Acute pyelonephritis with lesion of renal medullary necrosis |
| 590.2 | Renal perinephric abscess |
| 590.8 | Other pyelonephritis or pyonephrosis not specified as acute or chronic |
| 590.80 | Pyelonephritis, unspecified |
| 590.9 | Infection of kidney, unspecified |
| 595.0 | Acute cystitis |
| 595.89 | Other specified types of cystitis |
| 595.9 | Cystitis, unspecified |
| 599.0 | Urinary tract infection, site not specified (urosepsis could be included here) |

**International Statistical Classification of diseases and related health Problems 10th Revision. ICD-10. Version 2015.**
http://apps.who.int/classifications/icd10/browse/2015/en

| **ICD-10 CM Codes** | **Disease** |
| --- | --- |
| N10 | Acute tubulo-intersticial nephritis, including Acute Infectious interstitial nephritis, Acute pyelitis, and Acute pyelonephritis |
| N12 | Tubulo-interstitial nephritis, not specified as acute or chronic including Intersticial nephritis NOS, Pyelitis NOS, and Pyelonephritis NOS |
| N13.6 | Pyonephrosis, includes Obstructive uropathy with infection |
| N15.1 | Renal and perinephric abscess |
| N15.9 | Renal tubulo-intersticial disease, unspecified including Infection of kidney NOS |
| N30.0 | Acute cystitis |
| N30.8 | Other cystitis including Abscess of bladder |
| N30.9 | Cystitis, unspecified |
| N39.0 | Urinary tract infection, site not specified |

**Calculating the risk of Multidrug-Resistant (MDR) Gram-negative bacteria (GNB) infections in new patients with complicated urinary tract infections (UTIs)**

The probability of developing an MDR-GNB infection in a particular patient can be calculated as follows:

$$P\left( MDR \right)=\frac{1}{1+exp\left( -\sum\beta x \right)}$$

where $\left( \sum\beta x \right)$ is the linear function of key predictors in the model. Each predictor is multiplied by its corresponding as in the table below.


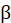


For illustrative purposes, we calculate here the MDR-GNB risk for a male, who acquired a cUTI in a medical facility, has an indwelling urinary catheter, has not had a UTI within the previous year, and has received antibiotic treatment within the previous 30 days.

| **Key Factor** | **Log-Odds** | **Patient** | **90% High** | **Average** | **10% Low** |
| --- | --- | --- | --- | --- | --- |
| (Intercept) | -2.31 | -- | -2.98 | -2.31 | -1.72 |
| Females/males | 0.51 | Males | 0.51 | 0.51×1=0.51 | 0.51 |
| Acquisition in a medical facility | 0.95 | Yes | 0.95 | 0.95×1=0.95 | 0.95 |
| Indwelling urinary catheter | 0.36 | Yes | 0.36 | 0.36×1=0.36 | 0.36 |
| UTI within the previous year | 0.64 | No | 0 | 0.64×0=0 | 0 |
| Antibiotic treatment within the previous 30 days | 0.52 | Yes | 0.52 | 0.52×1=0.52 | 0.52 |
| $\left( \sum\beta x \right)$ |  |  | -1.03 | 0.03 | 1.57 |
| $P\left( MDR \right)=\frac{1}{1+exp\left( -\sum\beta x \right)}$ |  |  | 0.65 | 0.49 | 0.35 |

Based on the calculation, the risk for such a patient having an MDR-GNB infection is **49%** when assisted in an average center, **35**% if in a center with low MDR incidence, and **65**% if in a center with high MDR incidence.
